# Avelumab in patients with previously treated metastatic melanoma: phase 1b results from the JAVELIN Solid Tumor trial

**DOI:** 10.1186/s40425-018-0459-y

**Published:** 2019-01-16

**Authors:** Ulrich Keilholz, Janice M. Mehnert, Sebastian Bauer, Hugues Bourgeois, Manish R. Patel, Donald Gravenor, John J. Nemunaitis, Matthew H. Taylor, Lucjan Wyrwicz, Keun-Wook Lee, Vijay Kasturi, Kevin Chin, Anja von Heydebreck, James L. Gulley

**Affiliations:** 1 Charité Comprehensive Cancer Center, Charitéplatz 1, 10117 Berlin, Germany; 2 Rutgers Cancer Institute of New Jersey, New Brunswick, NJ USA; 3 Department of Medical Oncology, University Hospital Essen, West German Cancer Center, University Duisburg-Essen, Essen, Germany; 4 Clinique Victor Hugo - Centre Jean Bernard, Le Mans, France; 5 Florida Cancer Specialists/Sarah Cannon Research Institute, Sarasota, FL USA; 6 Baptist Cancer Center, Memphis, TN USA; 7 University of Toledo College of Medicine, Toledo, OH USA; 8 Oregon Health & Science University, Portland, OR USA; 9 Maria Skłodowska-Curie Memorial Cancer Center, Department of Oncology and Radiotherapy and Biostatistics and Bioinformatics Unit, Warsaw, Poland; 10 Seoul National University Bundang Hospital, Seoul National University College of Medicine, Seongnam, South Korea; 11 EMD Serono, Billerica, MA USA; 12 EMD Serono, Rockland, MA USA; 13 Merck KGaA, Darmstadt, Germany; 14 Genitourinary Malignancies Branch and Laboratory of Tumor Immunology and Biology, Center for Cancer Research, National Cancer Institute, National Institutes of Health, Bethesda, MD USA

**Keywords:** PD-L1, Avelumab, Immune checkpoint inhibitor, Ocular melanoma, Cutaneous melanoma

## Abstract

**Background:**

We report phase 1b data from patients enrolled in the JAVELIN Solid Tumor clinical trial (NCT01772004) with unresectable stage IIIC or IV melanoma that had progressed after ≥1 line of therapy for metastatic disease.

**Patients and methods:**

Patients received avelumab (10 mg/kg)—a human anti–PD-L1 antibody. Assessments included objective response rate (ORR), progression-free survival (PFS), overall survival (OS), and safety.

**Results:**

As of December 31, 2016, 51 patients were treated and followed for a median of 24.2 months (range, 16.1–31.5). Most patients had cutaneous (*n* = 28 [54.9%]) or ocular (*n* = 16 [31.4%]) melanoma and had received a median of 2 prior lines of therapy (range, 0–4), including ipilimumab (*n* = 26 [51.0%]). The confirmed ORR was 21.6% (95% CI, 11.3–35.3; complete response, 7.8%; partial response, 13.7%). The median duration of response was not estimable (95% CI, 2.6 months-not estimable). Median PFS and OS were 3.1 months (95% CI, 1.4–6.3) and 17.2 months (95% CI, 6.6-not estimable), respectively. Subgroup analyses suggested meaningful clinical activity (ORR [95% CI]) in patients with non-ocular melanoma (31.4% [16.9–49.3]), PD-L1–positive tumors (42.1% [20.3–66.5]), or prior ipilimumab therapy (30.8% [14.3–51.8]). Thirty-nine patients (76.5%) had a treatment-related adverse event (TRAE), most commonly infusion-related reaction (29.4%), fatigue (17.6%), and chills (11.8%); 4 patients (7.8%) had a grade 3 TRAE. Five patients (9.8%) had an immune-related TRAE (all were grade 1/2). No grade 4 TRAEs or treatment-related deaths were reported.

**Conclusion:**

Avelumab showed durable responses, promising survival outcomes, and an acceptable safety profile in patients with previously treated metastatic melanoma.

**Trial registration:**

ClinicalTrials.gov identifier: NCT01772004.

**Electronic supplementary material:**

The online version of this article (10.1186/s40425-018-0459-y) contains supplementary material, which is available to authorized users.

## Introduction

Cutaneous melanoma (the most common melanoma subtype) is the 15th most prevalent cancer worldwide, with an estimated 232,000 diagnoses each year, and accounts for 1.6% of all cancers [1]. Non-cutaneous melanoma comprises less common, difficult-to-treat melanoma subtypes that occur on mucosal membranes of the head and neck and membranes lining the gastrointestinal and genitourinary tracts [2]. A very rare melanoma subtype arises at the uvea of the eye (also referred to as ocular melanoma). Most patients with cutaneous melanoma initially present with localized disease (84%), 9% with regional disease, and 4% with distant metastatic disease [3]. Patients with distant metastatic cutaneous melanoma have historically had poor prognoses (estimated 5-year survival rate of 17%), compared with 98% and 63% for patients with localized and regional disease, respectively, as shown in comprehensive analyses of US patient data collected between 2005 and 2011 [3]. However, long-term survival rates for patients with metastatic disease are improving with the incorporation of novel treatment options [4, 5], such as BRAF- and MEK-targeted therapies, intratumoral oncolytic herpes viral therapies, and immune checkpoint inhibitors (ICIs) targeting cytotoxic T-lymphocyte associated–protein 4 (CTLA-4) and programmed cell death-1 (PD-1) [6]. Notably, cutaneous melanoma is typically characterized by extensive tumor infiltration by T cells, high mutational burden, and an immunosuppressive phenotype, thereby supporting a role for ICIs. In contrast, the rarer, non-cutaneous subtypes are distinct from cutaneous melanoma with respect to presentation, staging, response to treatment, and patterns of progression [7–9]. Indeed, pembrolizumab, nivolumab, ipilimumab, and the combination of nivolumab and ipilimumab have demonstrated efficacy and are now approved by the United States Food and Drug Administration (FDA) and European Commission as treatment options for patients with advanced cutaneous melanoma [10–12].

Avelumab is a human anti–programmed death-ligand 1 (PD-L1) IgG1 monoclonal antibody that inhibits the PD-L1/PD-1 immune checkpoint [13]. Unlike other anti–PD-L1/PD-1 antibodies, avelumab contains a native Fc region and is capable of engaging natural killer cells to induce innate effector function against tumor cells, as shown in preclinical models [14, 15]; additional investigation is needed to determine the contribution of innate effector function to the overall antitumor response of avelumab. Avelumab is the first FDA- and European Commission—approved treatment option for patients with metastatic Merkel cell carcinoma—a rare and aggressive cutaneous malignancy that is the second most common cause of skin-cancer death after melanoma [16, 17]. Avelumab is also FDA-approved for the treatment of patients with locally advanced or metastatic urothelial carcinoma whose disease progressed during or following platinum-containing chemotherapy.

The safety and efficacy of avelumab has been investigated in the large, multicohort, phase 1 JAVELIN Solid Tumor clinical trial. In the phase 1a, dose-escalation part of the study, avelumab was safely administered at doses up to 20 mg/kg every 2 weeks (Q2W). Based on pharmacokinetic and pharmacodynamic data, avelumab 10 mg/kg Q2W was chosen for further investigation [13]. Avelumab has shown acceptable safety and durable antitumor activity in multiple tumor types investigated in the phase 1b, dose-expansion part of the study, including non-small cell lung cancer, urothelial carcinoma, and metastatic breast cancer [18–21]. Here, we report the safety and efficacy from a cohort of previously treated patients with locally advanced or metastatic melanoma enrolled in the phase 1b, dose-expansion part of the JAVELIN Solid Tumor trial with ≥16 months of follow-up.

## Materials and methods

### Study design and patients

JAVELIN Solid Tumor is an ongoing, international, multicenter, multicohort, open-label, dose-escalation and dose-expansion, phase 1 trial of avelumab in patients with advanced solid tumors (NCT01772004). In this phase 1b, dose-expansion cohort, eligible patients had histologically or cytologically confirmed stage IIIC or IV unresectable melanoma (according to the American Joint Committee on Cancer/Union for International Cancer Control [AJCC/UICC] TNM staging system, 7th edition) [22, 23] and were required to have progressive disease after ≥1 prior standard therapy for metastatic disease. Other eligibility criteria included an Eastern Cooperative Oncology Group performance status (ECOG PS) of 0 or 1; age≥18 years; adequate hematologic, hepatic, and renal function; no evidence of brain metastases; and an available fresh or archival tumor specimen. Patients were not selected based on tumor PD-L1 expression. Patients with ocular melanoma were permitted to be enrolled. Patients who received prior therapy with anti–PD-L1/PD-1 antibodies were excluded; however, patients who received prior therapy with anti–CTLA-4 antibodies were eligible. Other exclusion criteria included any previous anticancer treatment or major surgery ≤28 days before the start of study treatment; other cancer diagnosis ≤5 years prior to study entry; rapidly progressive disease; previous stem cell or solid organ transplant; known hypersensitivity to monoclonal antibodies; active or history of autoimmune disease or immunodeficiency; significant acute or chronic infection (e.g., human immunodeficiency virus, hepatitis B virus, hepatitis C virus); persisting toxicity related to prior therapy of grade >1 (except for grade 2 sensory neuropathy); and being pregnant or lactating. Any use of steroids was tapered before study treatment, except for patients with adrenal insufficiency—who could continue treatment at a physiological replacement dose.

This trial was conducted in accordance with the ethics principles of the Declaration of Helsinki and the International Council on Harmonization Guidelines on Good Clinical Practice. The protocol was approved in each center by the institutional review board or independent ethics committee. All patients provided written consent before their enrollment.

### Treatments and assessments

Avelumab 10 mg/kg was administered as a 1-h intravenous infusion Q2W until progression, unacceptable toxicity, or occurrence of any other protocol-specified criterion for withdrawal. Dose modifications were not permitted. The following adverse events (AEs) required treatment discontinuation: any grade 4 AE, except single laboratory values out of the normal range that were unrelated to study treatment, without clinical correlate, and resolved in ≤7 days with medical management; any grade ≥3 treatment-related amylase or lipase abnormality that was not associated with symptoms or clinical manifestations of pancreatitis and did not require dose delay; increased ECOG PS ≥3 that did not resolve to ≤2 by cycle day 14 of the following cycle (infusions were not given during the following cycle if the ECOG PS was ≥3 on the day of administration); or any grade 3 AE except for transient (≤6 h) influenza-like symptoms or fever controlled with medical management; fatigue, local infusion-related reaction (IRR), headache, nausea, or emesis that resolved to grade ≤ 1 in ≤24 h; single laboratory values out of the normal range that were unrelated to study treatment and without clinical correlate (excluding grade ≥3 increase in liver enzyme concentrations) that resolved to grade ≤1 in ≤7 days; and tumor flare (local pain, irritation, or localized rash at sites of known or suspected malignant tissue). Grade 2 AEs were managed via reductions in infusion rates and dose delays. AEs that did not resolve to grade ≤1 by the end of the next treatment cycle or that recurred, resulted in permanent withdrawal of avelumab (except for hormone insufficiencies that could be managed by replacement therapy). Premedication with an antihistamine and acetaminophen was administered 30 to 60 min prior to all infusions of avelumab.

Biweekly safety assessments included documentation of AEs and concurrent medications, and ECOG PS; other safety assessments were conducted every 6 weeks and included physical examinations and clinical laboratory tests (hematology and serum chemistry). AEs and laboratory abnormalities were classified and graded according to the National Cancer Institute Common Terminology Criteria for Adverse Events (NCI-CTCAE) version 4.0. A serious AE was defined as a life-threatening event that required hospitalization, resulted in disability, was a congenital anomaly, or resulted in death. IRRs (IRR, drug hypersensitivity, or hypersensitivity) occurring on the day of or the day after infusion and IRR symptoms occurring ≤1 day after infusion that resolved ≤2 days after onset were included. Immune-related AEs (irAEs) were identified using a prespecified list of AE terms and concomitant medication (eg, corticosteroids and hormone replacement) and relationship to study treatment was based on investigator assessment.

Clinical activity was assessed every 6 weeks by the investigators according to Response Evaluation Criteria in Solid Tumors (RECIST) version 1.1 [24]. Radiographic tumor assessments were performed at baseline and then every 6 weeks thereafter for the first 12 months, then every 12 weeks. For patients who had a partial response or complete response, a confirmatory computed tomography or magnetic resonance imaging scan was performed no sooner than 28 days later and preferably at the scheduled 6-week interval visit.

PD-L1 expression was assessed using a proprietary immunohistochemistry assay (Dako PD-L1 immunohistochemistry 73-10 pharmDx; Dako, Carpinteria, CA) [18–21]. In this study, PD-L1–positive status was defined prospectively using a cutoff of ≥1% of tumor cell membrane staining of any intensity; other PD-L1 cutoffs were also evaluated.

### Outcomes

The primary objectives of the JAVELIN Solid Tumor trial were to assess dose-limiting toxicities within the first 3 weeks of treatment in the dose-escalation part of the study and confirmed best overall response as adjudicated by an independent review committee in specified expansion cohorts (not including melanoma) [13]. Prespecified endpoints in the melanoma cohort included investigator-assessed confirmed best overall response per RECIST v1.1, progression-free survival (PFS) per RECIST v1.1, overall survival (OS), tumor PD-L1 expression, and safety. All subgroup analyses of patients with ocular/non-ocular melanoma and those who received prior ipilimumab therapy were exploratory. Changes in the sum of target lesion diameters from baseline were evaluated in patients with baseline tumor assessments and ≥1 postbaseline assessment.

### Statistical analysis

A sample size of 50 patients was planned to provide point estimates and 95% Clopper-Pearson CIs for an objective response rate (ORR) of 10% (95% CI, 3.3–21.8%) in the case of 5 responders, and of 20% (95% CI, 10.0–33.7%) in the case of 10 responders. Time-to-event endpoints were estimated with the Kaplan-Meier method, and CIs for the medians were calculated using the Brookmeyer-Crowley method. *P* values for association between categorical variables were determined using the Fisher exact test. Safety and clinical activity were analyzed in all patients who received ≥1 dose of avelumab.

## Results

### Baseline patient characteristics

As of December 31, 2016, 51 patients had received avelumab monotherapy (Table 1). Most patients had cutaneous melanoma (*n* = 28 [54.9%]), and 16 patients (31.4%) had ocular melanoma. Among all patients, 17 (33.3%) and 9 (17.7%) received 2 or ≥3 prior lines of therapy for metastatic or locally advanced disease, respectively; patients had received a median of 2 prior treatments (range, 0–4 treatments). Most patients (*n* = 26 [51.0%]) had received prior therapy with ipilimumab (anti–CTLA-4).

**Table 1:** Patient demographics and baseline characteristics

Characteristic	*N* = 51
Age, n (%)	
<65 years	28 (54.9)
≥65 years	23 (45.1)
Median (range), years	64.0 (31–84)
Sex, n (%)	
Male	34 (66.7)
Female	17 (33.3)
Race, n (%)	
White	35 (68.6)
Asian	2 (3.9)
American Indian or Alaskan	1 (2.0)
Black or African American	1 (2.0)
Other	12 (23.5)
ECOG performance status, n (%)	
0	25 (49.0)
1	26 (51.0)
Site of primary tumor, n (%)	
Cutaneous	28 (54.9)
Ocular	16 (31.4)
Mucosal	2 (3.9)
Other^a^	5 (9.8)
Time from initial diagnosis to study entry, years	
Median	4.3
Range	0.3–33.5
Time since first metastatic disease, months	
Median	14.8
Range	2.3–168.9
PD-L1 expression (≥1% of tumor cells), n (%)	
Positive	19 (37.3)
Negative	20 (39.2)
Not evaluable	12 (23.5)
Number of prior lines of therapy for metastatic or locally advanced disease, n (%)	
0	1 (2.0)
1	23 (45.1)
2	17 (33.3)
≥3	9 (18.0)
Missing	1 (2.0)
Median (range)	2 (0–4)
Prior anticancer therapy in >5% of patients, n (%)	
Ipilimumab	26 (51.0)
Dacarbazine	11 (21.6)
Cisplatin	8 (15.7)
Interferon	6 (11.8)
Fotemustine	5 (9.8)
Gemcitabine	5 (9.8)
Treosulfan	5 (9.8)
Vemurafenib	5 (9.8)
Investigational drug	4 (7.8)
Paclitaxel	3 (5.9)
Sorafenib	3 (5.9)

^a^ Includes melanoma of the canthus (*n* = 1) and unknown primary (*n* = 4)

At the time of data cutoff, the median duration of treatment with avelumab was 3.2 months (range, 0.5–27.2 months), and the median follow-up time was 24.2 months (range, 16.1–31.5 months). Patients had received a median of 7 doses of avelumab (range, 1–56 doses). At the time of analysis, treatment was ongoing in 6 patients (11.8%). Reasons for treatment discontinuation included progressive disease (*n* = 30 [58.8%]), AE (*n* = 8 [15.7%]), consent withdrawal (*n* = 3 [5.9%]), and death (*n* = 2 [3.9%]).

### Antitumor activity

**Fig. 1:**
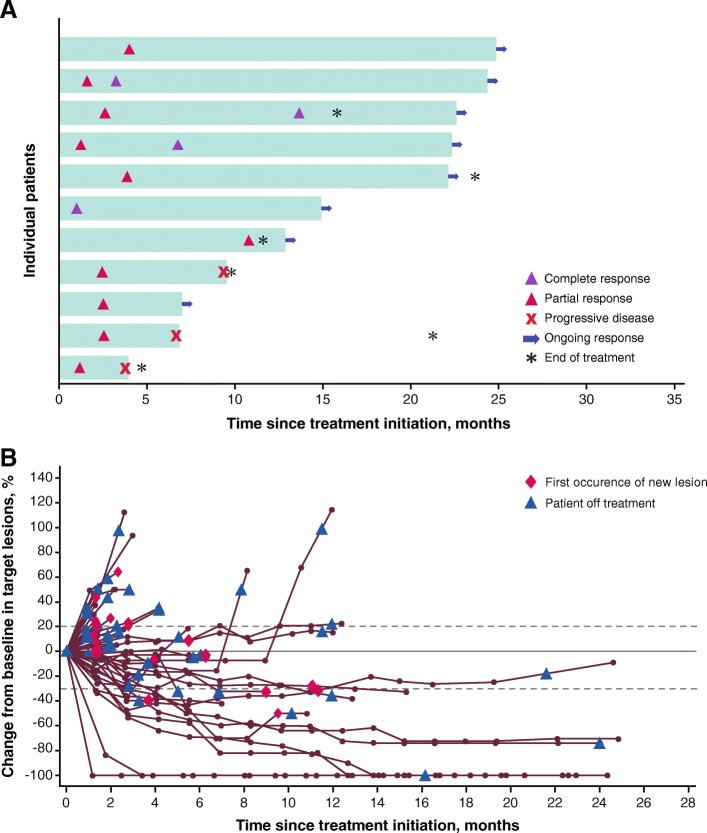
Time to and duration of response (**a**) and change in tumors in all patients (**b**)

Of all 51 patients, the confirmed ORR per RECIST v1.1 was 21.6% (95% CI, 11.3–35.3) (Table 2), with complete response in 4 patients (7.8%), partial response in 7 patients (13.7%), stable disease in 16 patients (31.4%), and progressive disease in 18 patients (35.3%); disease control was achieved in 52.9% of patients. Six patients (11.8%) were not evaluable for best overall response due to lack of available postbaseline assessments (*n* = 4), postbaseline assessments with an overall response that was non-evaluable (*n* = 1), or stable disease of insufficient duration (*n* = 1). Most responses occurred rapidly: of the 11 responses, 4 occurred by first postbaseline assessment (1 complete response and 3 partial responses), and 4 additional patients achieved a partial response by the second postbaseline assessment (Fig. 1a). Responses were ongoing in 8 of 11 responding patients (72.7%) at the cutoff date. The median duration of response (DOR) was not estimable (range, 2.6 months-not estimable). Based on Kaplan-Meier estimates, 80.0% (95% CI, 40.9–94.6) and 68.6% (95% CI, 30.5–88.7) of responding patients had DOR of 6 and 12 months, respectively. Of 45 patients with baseline and postbaseline assessments, 15 (33.3%) experienced tumor shrinkage of ≥30% (Fig. 1b).

**Table 2:** Response and outcomes in all patients and select patient subgroups

Outcome	All patients *N* = 51	Site of primary tumor		PD-L1 expression (≥1% of tumor cells)			Prior ipilimumab therapy	
		Non-ocular^a^ *n* = 35	Ocular *n* = 16	Positive *n* = 19	Negative *n* = 20	Not evaluable *n* = 12	Yes *n* = 26	No *n* = 25
ORR (95% CI), %	21.6 (11.3–35.3)	31.4 (16.9–49.3)	0 (0–20.6)	42.1 (20.3–66.5)	0 (0–16.8)	25.0 (5.5–57.2)	30.8 (14.3–51.8)	12.0 (2.5–31.2)
PFS								
Median (95% CI), months	3.1 (1.4–6.3)	3.9 (2.0–9.0)	1.7 (1.4–4.1)	6.3 (2.1–11.1)	1.4 (1.3–4.1)	3.3 (1.4-NE)	6.3 (1.4–9.5)	2.8 (1.4–4.1)
6-month rate (95% CI), %	39.2 (25.2–52.9)	47.1 (28.7–63.4)	23.4 (6.5–46.3)	52.6 (28.7–71.9)	22.4 (6.2–44.7)	40.0 (12.3–67.0)	50.3 (29.3–68.0)	26.6 (10.4–46.1)
12-month rate (95% CI), %	17.4 (7.8–30.0)	25.9 (11.8–42.5)	0 (NE-NE)	24.1 (7.8–45.1)	0 (NE-NE)	30.0 (7.1–57.8)	23.3 (8.7–41.9)	10.7 (1.9–28.3)
OS								
Median (95% CI), months	17.2 (6.6-NE)	17.2 (9.3-NE)	NE (3.6-NE)	24.9 (6.2-NE)	5.3 (3.8–16.2)	NE (2.1-NE)	16.2 (5.3-NE)	17.2 (4.7-NE)
6-month rate (95% CI), %	68.7 (52.9–80.2)	75.8 (55.6–87.7)	56.3 (29.5–76.2)	78.9 (53.2–91.5)	44.7 (20.5–66.5)	88.9 (43.3–98.4)	70.8 (48.4–84.9)	66.9 (42.5–82.8)
12-month rate (95% CI), %	59.4 (43.4–72.2)	64.9 (44.5–79.4)	50.0 (24.5–71.0)	68.4 (42.8–84.4)	37.3 (14.8–60.1)	77.8 (36.5–93.9)	62.5 (40.3–78.4)	56.1 (32.2–74.5)
24-month rate (95% CI), %	43.7 (27.3–58.9)	44.8 (25.5–62.4)	NE	54.1 (27.4–74.7)	NE	64.8 (25.3–87.2)	49.2 (28.1–67.3)	NE

^a^ Patients with cutaneous or mucosal melanoma, melanoma of the canthus (*n* = 1), and unknown primary (*n* = 4)

*NE* not estimable

**Fig. 2:**
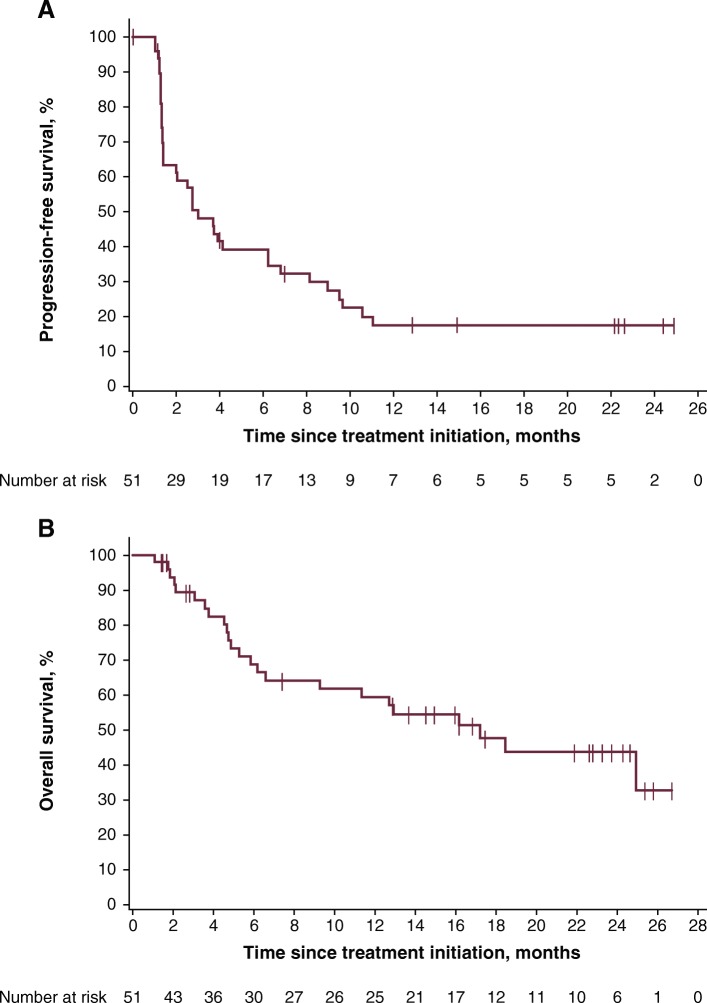
PFS (**a**) and OS (**b**) in all patients

Median PFS was 3.1 months (95% CI, 1.4–6.3) (Table 2), and 14 patients (27.5%) were event-free at the cutoff date. The 6- and 12-month rates of PFS were 39.2 and 17.4%, respectively. The PFS curve demonstrated a stable plateau following the 12-month time point (Fig. 2a).

Median OS was 17.2 months (95% CI, 6.6-not estimable) (Table 2 and Fig. 2b), and 27 patients (52.9%) were alive at the cutoff date. The 12- and 24-month rates of OS were 59.4% and 43.7%, respectively.

### Subgroup analyses

Of 35 patients with cutaneous or mucosal melanoma, melanoma of the canthus, or unknown primary (collectively referred to as patients with non-ocular melanoma), the ORR was 31.4% (95% CI, 16.9–49.3) (Table 2), which included the previously described 11 patients with an objective response. ORRs according to tumor site subgroups are shown in Additional file 1: Table S1.

**Fig. 3:**
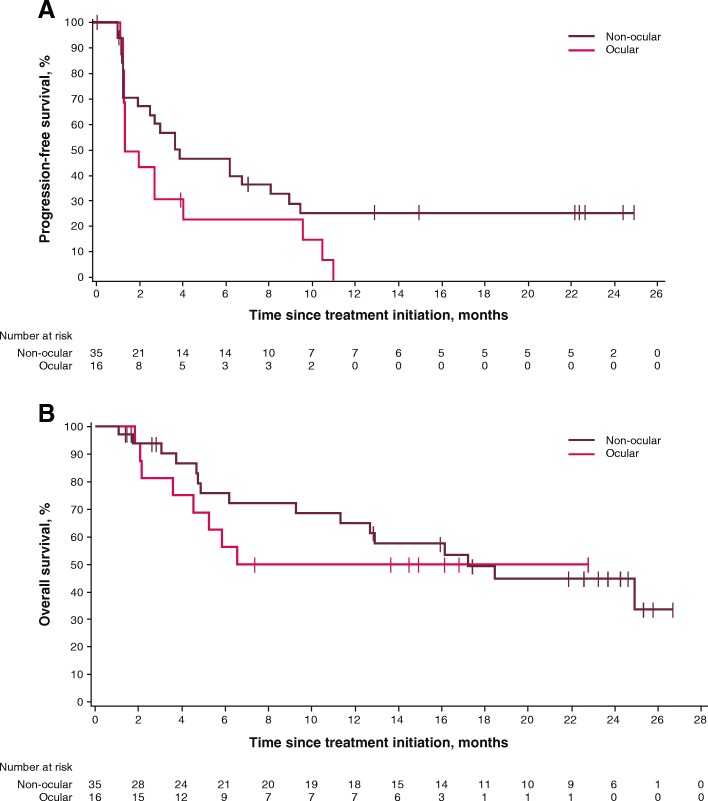
PFS (**a**) and OS (**b**) in patients with non-ocular and ocular melanoma

In patients with non-ocular melanoma, median PFS was 3.9 months (95% CI, 2.0–9.0), and 6- and 12-month PFS rates were 47.1 and 25.9%, respectively. Median OS was 17.2 months (95% CI, 9.3-not estimable), and 12- and 24-month OS rates were 64.9% and 44.8%, respectively. No objective responses were observed in the 16 patients with ocular melanoma, although 7 of 16 patients (43.8%) had transient stable disease. Median PFS was 1.7 months (95% CI, 1.4–4.1), with 6- and 12-month rates of PFS of 23.4 and 0%, respectively. Median OS was not yet reached (95% CI, 3.6 months-not estimable); the 12-month OS rate was 50.0% and the 24-month OS rate was not estimable (Table 2**,** Fig. 3**,** and Additional file 2: Figure S1).

Of 39 patients with biopsy material assessable for PD-L1 expression, those with PD-L1–positive tumors at a 1% cutoff (*n* = 19) had a confirmed ORR of 42.1% (95% CI, 20.3–66.5): 2 patients experienced a complete response and 6 a partial response vs an ORR of 0% (95% CI, 0–16.8) in patients with PD-L1–negative tumors (*n* = 20) (Fisher exact test, *P* = 0.001); in patients whose tumors were not evaluable for PD-L1 expression (*n* = 12), the ORR was 25.0% (95% CI, 5.5–57.2): 2 patients experienced a complete response and 1 a partial response (Table 2 and Additional file 2: Figure S1). If confined to patients with non-ocular melanoma, objective responses were observed in 8 of 14 patients (57.1% [95% CI, 28.9–82.3]) with PD-L1–positive tumors vs an ORR of 0% (95% CI, 0–26.5) in patients with PD-L1–negative tumors (*n* = 12) (Fisher exact test, *P* = 0.002); of 9 patients whose tumors were not evaluable for PD-L1 expression, 3 objective responses were observed (33.3% [95% CI, 7.5–70.1]) (Additional file 3: Table S2).

**Fig. 4:**
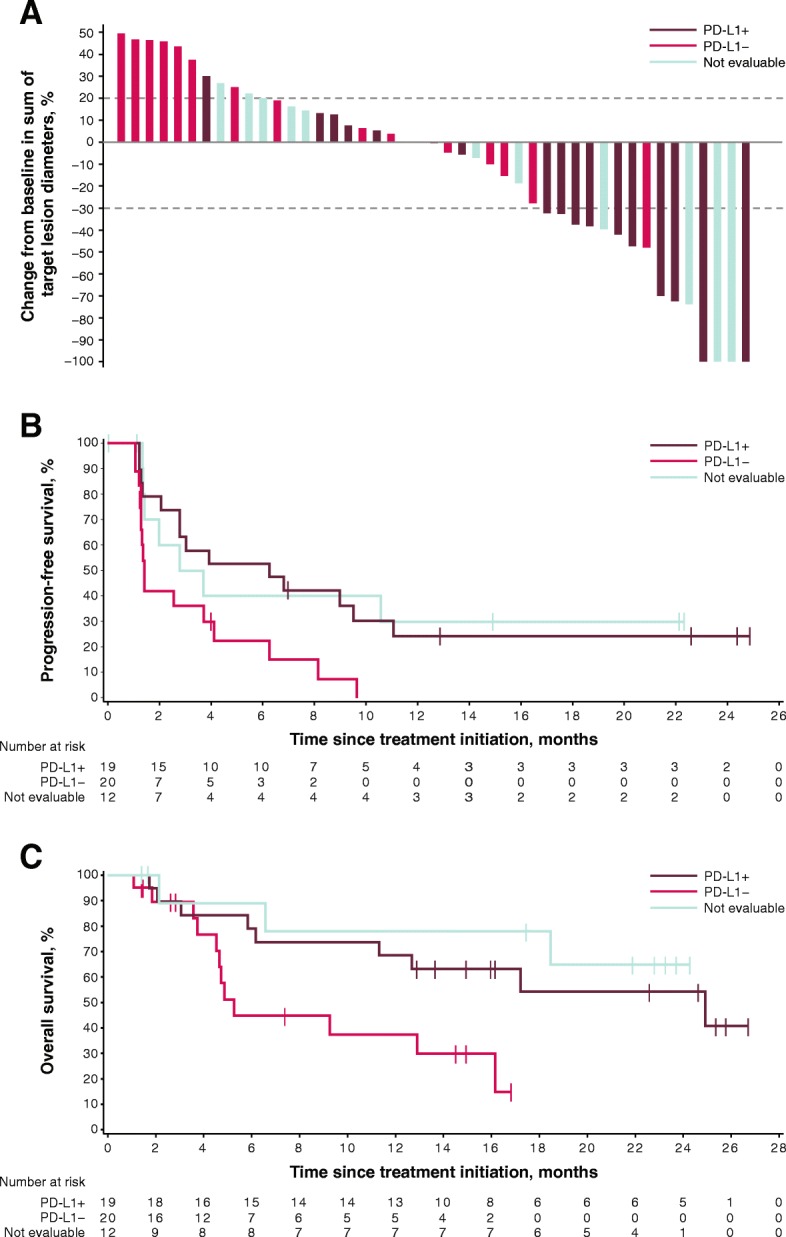
Best change in sum of target lesion diameters from baseline (**a**), and PFS (**b**) and OS (**c**) according to tumor PD-L1 expression status at 1% cutoff in evaluable patients

Of 45 patients with baseline and postbaseline measurements, 23 patients had a reduction in the sum of target lesion diameters of any kind: 12 patients with PD-L1–positive tumors, 5 with PD-L1–negative tumors, and 6 patients whose tumors were not evaluable for PD-L1 expression (Fig. 4a). Of these patients, tumor shrinkage was ≥30% in 10 patients with PD-L1–positive tumors, in 1 patient with a PD-L1–negative tumor, and in 4 patients with tumors not evaluable for PD-L1 expression. The changes in the sums of target lesions between baseline and best postbaseline assessment according to tumor site are shown in Additional file 4: Figure S2.

Median PFS was 6.3 months (95% CI, 2.1–11.1; HR, 0.41 [95% CI, 0.20–0.86]) in patients with PD-L1–positive tumors, 1.4 months (95% CI, 1.3–4.1) in those with PD-L1–negative tumors, and 3.3 months (95% CI, 1.4-not estimable; HR, 0.39 [95% CI, 0.16–0.97]) in those with tumors not evaluable for PD-L1 expression (Table 2 and Fig. 4b). Patients with PD-L1–positive tumors had a median OS of 24.9 months (95% CI, 6.2-not estimable; HR, 0.34 [95% CI, 0.13–0.87]) compared with 5.3 months (95% CI, 3.8–16.2) in those with PD-L1–negative tumors; median OS was not estimable (95% CI, 2.1-not estimable; HR, 0.21 [95% CI, 0.05–0.79]) in patients whose tumors were not evaluable for PD-L1 expression (Table 2 and Fig. 4c). If confined to patients with non-ocular melanoma, median PFS was 7.9 months (95% CI, 1.3-not estimable) in patients with PD-L1–positive tumors, 1.3 months (95% CI, 1.1–6.3) in patients with PD-L1–negative tumors, and 3.7 months (95% CI, 1.4-not estimable) in patients whose tumors were not evaluable for PD-L1 expression. Median OS was 24.9 months (95% CI, 6.2-not estimable), 4.9 months (95% CI, 3.8–12.9), and not estimable (95% CI, 18.5-not estimable), respectively (Additional file 3: Table S2 and Additional file 5: Figure S3).

The confirmed ORR was 30.8% (95% CI, 14.3–51.8) in patients who received prior ipilimumab therapy (*n* = 26), compared with 12.0% (95% CI, 2.5–31.2) in patients who did not (*n* = 25) (Table 2 and Additional file 2: Figure S1). Median PFS of 6.3 months (95% CI, 1.4–9.5) in patients who received prior ipilimumab therapy compared with 2.8 months (95% CI, 1.4–4.1) in patients who did not. Median OS was 16.2 months (95% CI, 5.3 months-not estimable) in patients who received prior ipilimumab therapy compared with 17.2 months (95% CI, 4.7 months-not estimable) in patients who did not (Table 2).

ORRs for other subgroups can be found in Additional file 2: Figure S1.

### Safety

Overall, 50 patients (98.0%) had an AE (Additional file 6: Table S3), 39 (76.5%) of whom had a treatment-related AE (TRAE) of any grade (Table 3). The most common TRAEs (occurring in >10% of patients) were IRR (*n*= 15 [29.4%]), fatigue (*n*= 9 [17.6%]), and chills (*n*= 6 [11.8%]). Grade 3 TRAEs occurred in 4 patients (7.8%): nausea, gamma-glutamyltransferase increased, hypokalemia, and lipase increased (*n* = 1 [2.0%] each). A TRAE led to permanent discontinuation in 5 patients (9.8%)—most commonly IRR (*n* = 3 [5.9%]), nausea (*n* = 1 [2.0%]), and sarcoidosis (*n* = 1 [2.0%]). Serious TRAEs occurred in 3 patients (5.9%): IRR (resolved with concomitant medication), pyrexia (resolved with concomitant medication), and sarcoidosis (led to treatment discontinuation). All serious AEs of any causality are shown in Additional file 7: Table S4. No grade 4 TRAEs and no treatment-related deaths were reported.

**Table 3:** Incidence of treatment-related adverse events

Any-grade in ≥ 5% of patients or any grade 3 TRAEs	*N* = 51	
	Any grade n (%)	Grade 3 n (%)
Patients with ≥1 event	39 (76.5)	4 (7.8)
Infusion-related reaction	15 (29.4)	0
Fatigue	9 (17.6)	0
Chills	6 (11.8)	0
Diarrhea	5 (9.8)	0
Dysgeusia	4 (7.8)	0
Pyrexia	4 (7.8)	0
Aspartate aminotransferase increased	3 (5.9)	0
Dry mouth	3 (5.9)	0
Nausea	2 (3.9)	1 (2.0)
Gamma-glutamyltransferase increased	1 (2.0)	1 (2.0)
Hypokalemia	1 (2.0)	1 (2.0)
Lipase increased	1 (2.0)	1 (2.0)
All immune-related TRAEs		
Patients with ≥1 event	5 (9.8)	0
Hypothyroidism	2 (3.9)	0
Pneumonitis	2 (3.9)	0
Hyperthyroidism	1 (2.0)	0
Sarcoidosis	1 (2.0)	0
Vitiligo	1 (2.0)	0

IRRs occurred at the first infusion in 8 patients (15.7%), at the second infusion in 3 patients (5.9%), at the third infusion in 1 patient (2.0%), and at the fourth or later infusion in 3 patients (5.9%). IRRs led to treatment discontinuation in 3 patients (5.9%). Five patients (9.8%) experienced irAEs related to treatment, and all were grade 1/2: hypothyroidism and pneumonitis (*n* = 2 [3.9%] each) and hyperthyroidism, sarcoidosis, and vitiligo (*n* = 1 [2.0%] each) (Table 3). An irAE led to treatment discontinuation in 1 patient (2.0%) due to sarcoidosis (previously mentioned serious, treatment-related event).

## Discussion

In this analysis of previously treated patients with advanced melanoma, avelumab demonstrated durable responses and promising survival outcomes. Of all enrolled patients, the confirmed ORR was 21.6 and 31.4% in patients with non-ocular melanoma. These findings are consistent with results from larger pivotal studies of ICIs for second-line or later treatment of advanced melanoma, which ranged from 21 to 37% [25–30]. After a median follow-up of ≈2 years, the median DOR for avelumab was not estimable in this cohort of patients, consistent with findings after longer-term follow-up in pivotal studies of other ICIs [26, 28, 30]. Median PFS was 3.1 months and 3.9 months in patients with non-ocular melanoma, which was also comparable to that seen in these earlier pivotal studies [25–30], and ongoing clinical benefit was observed in a subset of patients—as evidenced by the plateau of the PFS curve. Although the number of patients who had received prior ipilimumab therapy in this analysis is small, the median OS of 16.2 months is comparable to that reported in studies of patients with advanced melanoma who received pembrolizumab (KEYNOTE-002; 13.4 months [2 mg/kg; *n* = 180] and 14.7 months [10 mg/kg; *n* = 181]) or nivolumab (CheckMate 037; 15.7 months [*n* = 272]) following disease progression on ipilimumab [26, 28]. Additionally, in a study of patients treated with pembrolizumab who had received ≤1 prior therapy that did not include an ICI (KEYNOTE-006), median OS had not been reached after a median follow up of 22.9 months [30].

Subgroup analyses suggested meaningful clinical activity in patients who had non-ocular primary tumors, received prior ipilimumab therapy, or had PD-L1–positive tumors. In the JAVELIN Solid Tumor melanoma cohort, no objective responses were observed in patients with ocular melanoma, which is consistent with reported ocular melanoma studies of other checkpoint inhibitors. However, 7 of 16 patients (43.8%) with ocular melanoma in this cohort had a best overall response of stable disease with avelumab, which may be explained by the minimal mutational load associated with ocular melanoma [31]. Despite a lack of objective response in these patients, OS appeared comparable to that of patients with non-ocular melanoma. Importantly, CheckMate 037, KEYNOTE-002, and KEYNOTE-006 did not enroll patients with ocular melanoma. In patients who received prior ipilimumab therapy, the confirmed ORR was 30.8%, which was consistent with that seen in ipilimumab-refractory patients receiving nivolumab (27%) or pembrolizumab (22% [2 mg/kg] and 28% [10 mg/kg]) [26, 28].

In patients evaluable for response according to PD-L1 expression, those with PD-L1–positive tumors had an ORR of 42.1%—57.1% when confined to patients with non-ocular melanoma—consistent with previous findings from CheckMate 037 (43.6%) [25, 29, 30]. In this study, patients with PD-L1–negative tumors did not achieve an objective response, in contrast with the observed modest efficacy in the much larger cohorts of patients with PD-L1–negative tumors in pivotal studies of ICIs [25–30]. However, a comparative study using samples obtained from patients with non-small cell lung cancer showed greater sensitivity of the PD-L1 immunohistochemistry assay used in this study (Dako 73–10) compared with that used in the KEYNOTE 002 and 006 studies (Dako 22C3) [32], suggesting a possible discrepancy in the ability to identify truly PD-L1–negative tumors. Although prior post hoc analyses assessed the association between response to nivolumab and PD-L1 expression across the full range of expression levels and suggested that ORR increased with increasing PD-L1 expression, no PD-L1 expression threshold that may predict response to nivolumab was identified [26]. Future research could endeavor to address whether additional biomarkers, such as tumor mutational burden [33–35], may identify subgroups of patients with either ocular or PD-L1–negative tumors who respond to avelumab.

The safety profile was considered manageable and tolerable, and generally consistent with that of other ICIs and of studies of avelumab monotherapy in advanced cancers [18–20]. In this study, grade 3 TRAEs occurred in 7.8% of patients and no grade 4 events and no treatment-related deaths were reported, compared with grade 3/4 TRAEs occurring in ≈15% of patients in studies of pembrolizumab and nivolumab, and few, but notable, reported deaths [26, 28, 30]. In addition, no grade 3/4 irAEs occurred compared with an incidence of ≈4% to 10% in these other studies [29, 31].

## Conclusions

Avelumab showed durable responses, promising survival outcomes, and an acceptable safety profile in patients with previously treated metastatic melanoma, consistent with other ICIs. Encouraging efficacy outcomes were observed in patients with PD-L1–positive tumors and in patients who had progressed on prior ipilimumab therapy.

## Additional files

Additional file 1: **Table S1.** ORR according to tumor site subgroups. (PDF 31 kb)

Additional file 2: **Figure S1.** Subgroup analyses of efficacy. (PDF) (PDF 135 kb)

Additional file 3: **Table S2.** Response and outcomes in patients with non-ocular melanoma according to PD-L1 status. (PDF) (PDF 319 kb)

Additional file 4: **Figure S2.** Best percentage change from baseline in target lesions in all evaluable patients (*n* = 45). (PDF 137 kb)

Additional file 5: **Figure S3.** PFS (A) and OS (B) in patients with non-ocular melanoma according to tumor PD-L1 expression (*n* = 35). (PDF) (PDF 111 kb)

Additional file 6: **Table S3.** All adverse events (*N* = 51). (PDF) (PDF 154 kb)

Additional file 7: **Table S4.** All serious adverse events (*N* = 51). (PDF) (PDF 101 kb)

## Abbreviations

AE: Adverse event
AJCC: American Joint Committee on Cancer
CI: Confidence interval
CTLA-4: Cytotoxic T-lymphocyte associated-protein 4
DOR: Duration of response
ECOG PS: Eastern Cooperative Oncology Group performance status
FDA: Food and Drug Administration
HR: Hazard ratio
ICI: Immune checkpoint inhibitor
IgG1: Immunoglobulin G1
irAE: Immune-related AE
IRR: Infusion-related reaction
NCI-CTCAE: National Cancer Institute Common Terminology Criteria for Adverse Events
NE: Not estimable
ORR: Objective response rate
OS: Overall survival
PD-1: Programmed cell death-1
PD-L1: Programmed death-ligand 1
PFS: Progression-free survival
Q2W: every 2 weeks
RECIST: Response Evaluation Criteria in Solid Tumors
TRAE: Treatment-related adverse event
UICC: Union for International Cancer Control
