# Supplementary material for: Avelumab in patients with previously treated metastatic melanoma: phase 1b results from the JAVELIN Solid Tumor trial
Source: J Immunother Cancer. 2019 Jan 16;7:12. doi: 10.1186/s40425-018-0459-y (PMC6335739; doi:10.1186/s40425-018-0459-y)
Supplement: Supplementary file 1 — Table S1. ORR according to tumor site subgroups. (PDF 31 kb) [file 40425_2018_459_MOESM1_ESM.pdf]

Additional file 1: Table S1. ORR according to tumor site subgroups

Site of primary tumor	n/N	ORR (95% CI)
Non-ocular	11/35	31.4 (16.9-49.3)
Cutaneous	8/28	28.6 (13.2-48.7)
Mucosal	1/2	50.0 (1.3-98.7)
Other[a]	2/5	40.0 (5.3-85.3)
Ocular	0/16	0 (0-20.6)

[a] Includes melanoma of the canthus (n=1) and unknown primary (n=4).
