# Supplementary material for: Avelumab in patients with previously treated metastatic melanoma: phase 1b results from the JAVELIN Solid Tumor trial
Source: J Immunother Cancer. 2019 Jan 16;7:12. doi: 10.1186/s40425-018-0459-y (PMC6335739; doi:10.1186/s40425-018-0459-y)
Supplement: Supplementary file 2 — Figure S1. Subgroup analyses of efficacy. (PDF) (PDF 135 kb) [file 40425_2018_459_MOESM2_ESM.pdf]

Additional file 2: Figure S1. Subgroup analyses of efficacy.

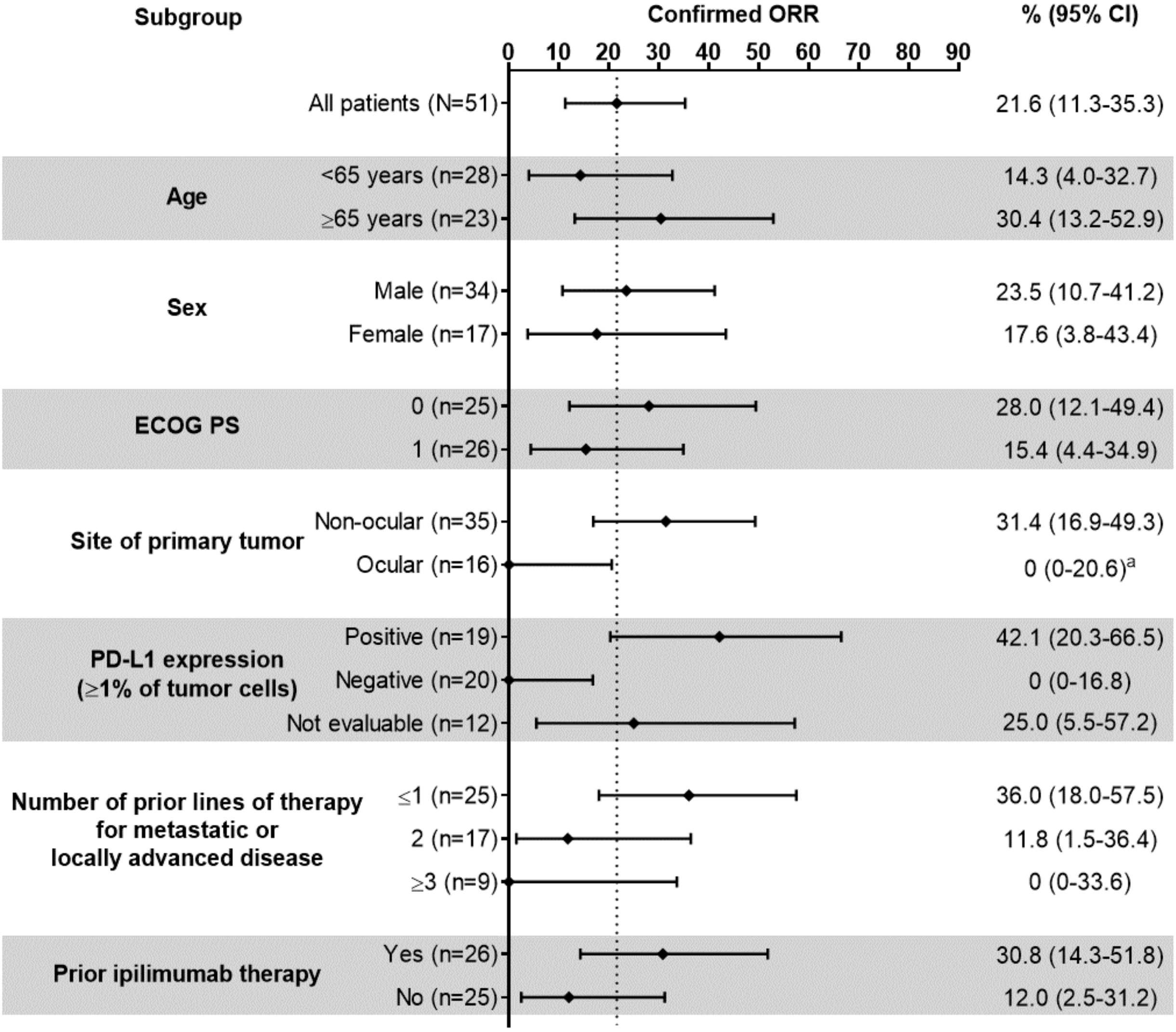

The dotted vertical line represents the ORR of the overall patient population.

[a] Of 13 patients evaluable for PD-L1 expression, 5 patients had PD-L1–positive tumors and 8 had PD-L1–negative tumors (1% cutoff).
