# Supplementary material for: Avelumab in patients with previously treated metastatic melanoma: phase 1b results from the JAVELIN Solid Tumor trial
Source: J Immunother Cancer. 2019 Jan 16;7:12. doi: 10.1186/s40425-018-0459-y (PMC6335739; doi:10.1186/s40425-018-0459-y)
Supplement: Supplementary file 3 — Table S2. Response and outcomes in patients with non-ocular melanoma according to PD-L1 status. (PDF) (PDF 319 kb) [file 40425_2018_459_MOESM3_ESM.pdf]

Additional file 3: Table S2. Response and outcomes in patients with non-ocular melanoma according to PD-L1 status.

Patients with non-ocular melanoma (n=35)			
**Outcome**	**PD-L1 expression (≥1% of tumor cells)**		
	**Positive n=14**	**Negative n=12**	**Not evaluable n=9**
ORR (95% CI), %	57.1 (28.9-82.3)	0 (0-26.5)	33.3 (7.5-70.1)
PFS			
Median (95% CI), months	7.9 (1.3-NE)	1.3 (1.1-6.3)	3.7 (1.4-NE)
6-month rate (95% CI), %	64.3 (34.3-83.3)	23.3 (3.6-52.9)	42.9 (9.8-73.4)
12-month rate (95% CI), %	33.3 (10.9-58.0)	0 (NE-NE)	42.9 (9.8-73.4)
OS			
Median (95% CI), months	24.9 (6.2-NE)	4.9 (3.8-12.9)	NE (18.5-NE)
6-month rate (95% CI), %	85.7 (53.9-96.2)	39.3 (9.3-69.3)	100 (NE-NE)
12-month rate (95% CI), %	71.4 (40.6-88.2)	26.2 (3.9-57.5)	100 (NE-NE)
24-month rate (95% CI), %	55.1 (25.5-77.1)	0 (NE-NE)	80.0 (20.4-96.9)
