# Supplementary material for: Avelumab in patients with previously treated metastatic melanoma: phase 1b results from the JAVELIN Solid Tumor trial
Source: J Immunother Cancer. 2019 Jan 16;7:12. doi: 10.1186/s40425-018-0459-y (PMC6335739; doi:10.1186/s40425-018-0459-y)
Supplement: Supplementary file 4 — Figure S2. Best percentage change from baseline in target lesions in all evaluable patients (n = 45). (PDF 137 kb) [file 40425_2018_459_MOESM4_ESM.pdf]

Additional file 4: Figure S2. Best percentage change from baseline in target lesions in all evaluable patients (n=45).[a]

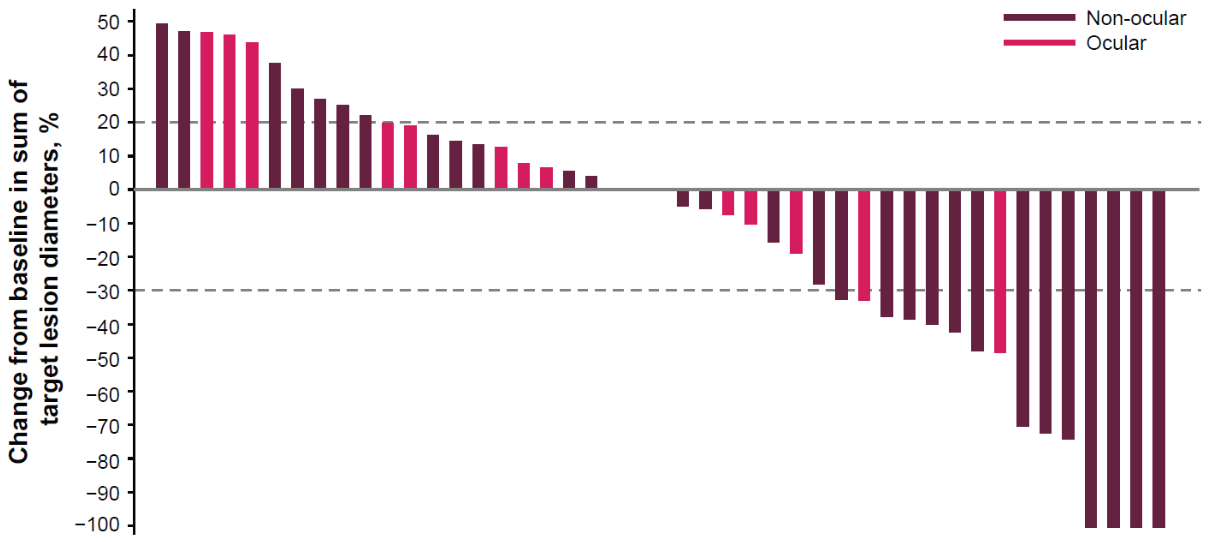

[a] Only patients with baseline and ≥1 postbaseline lesion assessment are included.
