# Supplementary material for: Avelumab in patients with previously treated metastatic melanoma: phase 1b results from the JAVELIN Solid Tumor trial
Source: J Immunother Cancer. 2019 Jan 16;7:12. doi: 10.1186/s40425-018-0459-y (PMC6335739; doi:10.1186/s40425-018-0459-y)
Supplement: Supplementary file 5 — Figure S3. PFS (A) and OS (B) in patients with non-ocular melanoma according to tumor PD-L1 expression (n = 35). (PDF) (PDF 111 kb) [file 40425_2018_459_MOESM5_ESM.pdf]

Additional file 5: Figure S3. PFS (A) and OS (B) in patients with non-ocular melanoma according to tumor PD-L1 expression (n=35).

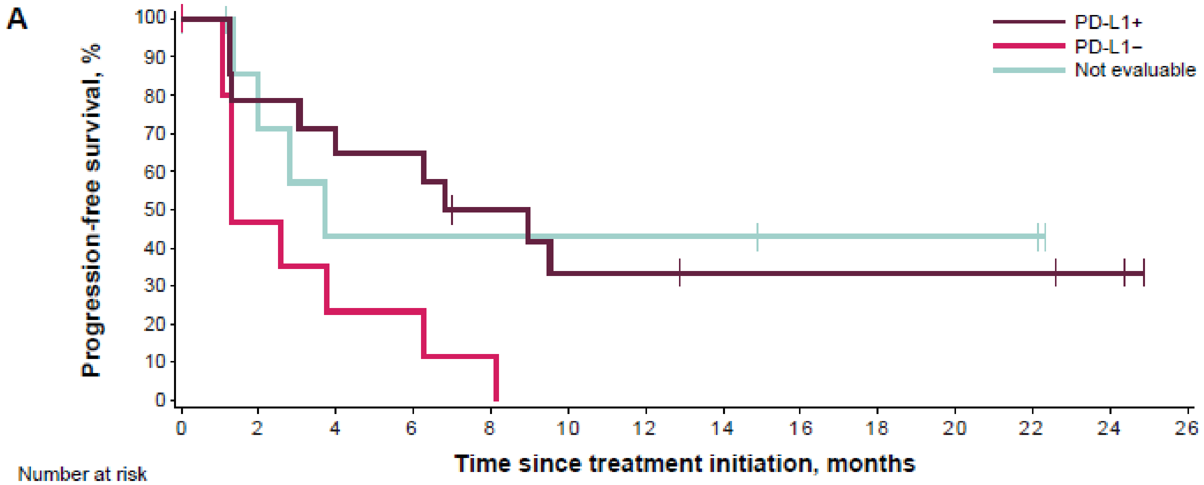

Number at risk

PD-L1+	14	11	9	9	6	4	4	3	3	3	3	3	2	0
PD-L1−	12	4	2	2	1	0	0	0	0	0	0	0	0	0
Not evaluable	9	6	3	3	3	3	3	3	2	2	2	2	0	0

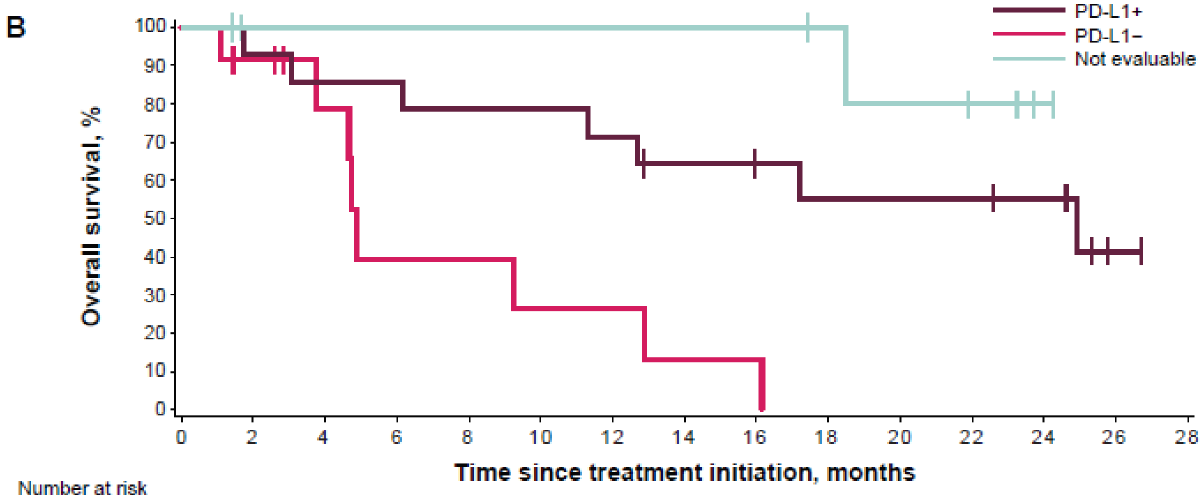

Number at risk

PD-L1+	14	13	12	12	11	11	10	8	7	6	6	6	5	1	0
PD-L1−	12	9	6	3	3	2	2	1	1	0	0	0	0	0	0
Not evaluable	9	6	6	6	6	6	6	6	6	5	4	3	1	0	0
