# Supplementary material for: Avelumab in patients with previously treated metastatic melanoma: phase 1b results from the JAVELIN Solid Tumor trial
Source: J Immunother Cancer. 2019 Jan 16;7:12. doi: 10.1186/s40425-018-0459-y (PMC6335739; doi:10.1186/s40425-018-0459-y)
Supplement: Supplementary file 6 — Table S3. All adverse events (N = 51). (PDF) (PDF 154 kb) [file 40425_2018_459_MOESM6_ESM.pdf]

**Additional file 6: Table S3.** All adverse events (N=51).

| Primary system organ class<br>MedDRA preferred term | All causality | | Treatment-related | |
|---|---|---|---|---|
| | Any grade, n (%) | Grade ≥3, n (%) | Any grade, n (%) | Grade ≥3, n (%) |
| Patients with ≥1 event | 50 (98.0) | 31 (60.8) | 39 (76.5) | 4 (7.8) |
| General disorders and administration site conditions | | | | |
| Fatigue | 11 (21.6) | 1 (2.0) | 9 (17.6) | 0 |
| Asthenia | 8 (15.7) | 3 (5.9) | 2 (3.9) | 0 |
| Chills | 8 (15.7) | 0 | 6 (11.8) | 0 |
| Edema peripheral | 7 (13.7) | 1 (2.0) | 2 (3.9) | 0 |
| Pyrexia | 7 (13.7) | 0 | 4 (7.8) | 0 |
| Disease progression | 5 (9.8) | 4 (7.8) | 0 | 0 |
| General physical health deterioration | 2 (3.9) | 1 (2.0) | 0 | 0 |
| Influenza-like illness | 2 (3.9) | 0 | 1 (2.0) | 0 |
| Malaise | 2 (3.9) | 0 | 0 | 0 |
| Peripheral swelling | 2 (3.9) | 0 | 0 | 0 |
| Catheter site hematoma | 1 (2.0) | 0 | 0 | 0 |
| Catheter site pain | 1 (2.0) | 0 | 0 | 0 |
| Catheter site swelling | 1 (2.0) | 0 | 0 | 0 |
| Chest pain | 1 (2.0) | 0 | 0 | 0 |
| Extravasation | 1 (2.0) | 0 | 0 | 0 |
| Gait disturbance | 1 (2.0) | 0 | 0 | 0 |
| Infusion site swelling | 1 (2.0) | 0 | 0 | 0 |
| Localized edema | 1 (2.0) | 0 | 0 | 0 |
| Noncardiac chest pain | 1 (2.0) | 0 | 0 | 0 |
| Gastrointestinal disorders | | | | |
| Diarrhea | 13 (25.5) | 0 | 5 (9.8) | 0 |
| Constipation | 10 (19.6) | 0 | 1 (2.0) | 0 |
| Nausea | 9 (17.6) | 2 (3.9) | 2 (3.9) | 1 (2.0) |
| Abdominal pain | 6 (11.8) | 1 (2.0) | 2 (3.9) | 0 |
| Dry mouth | 6 (11.8) | 0 | 3 (5.9) | 0 |
| Vomiting | 6 (11.8) | 1 (2.0) | 1 (2.0) | 0 |
| Ascites | 3 (5.9) | 1 (2.0) | 0 | 0 |
| Dysphagia | 3 (5.9) | 0 | 0 | 0 |
| Dyspepsia | 2 (3.9) | 0 | 0 | 0 |
| Gastrointestinal hemorrhage | 2 (3.9) | 2 (3.9) | 0 | 0 |
| Esophagitis | 2 (3.9) | 1 (2.0) | 0 | 0 |
| Abdominal pain lower | 1 (2.0) | 0 | 0 | 0 |
| Abdominal pain upper | 1 (2.0) | 0 | 0 | 0 |
| Anorectal discomfort | 1 (2.0) | 0 | 0 | 0 |
| Flatulence | 1 (2.0) | 0 | 1 (2.0) | 0 |
| Gastroesophageal reflux disease | 1 (2.0) | 0 | 0 | 0 |
| Glossodynia | 1 (2.0) | 0 | 0 | 0 |
| Portal hypertensive gastropathy | 1 (2.0) | 0 | 0 | 0 |
| Stomatitis | 1 (2.0) | 0 | 0 | 0 |
| Varices esophageal | 1 (2.0) | 0 | 0 | 0 |

| | | | | |
|---|---|---|---|---|
| Musculoskeletal and connective tissue disorders | | | | |
| Arthralgia | 7 (13.7) | 0 | 2 (3.9) | 0 |
| Back pain | 6 (11.8) | 1 (2.0) | 1 (2.0) | 0 |
| Musculoskeletal pain | 5 (9.8) | 0 | 0 | 0 |
| Musculoskeletal chest pain | 4 (7.8) | 0 | 1 (2.0) | 0 |
| Myalgia | 4 (7.8) | 0 | 2 (3.9) | 0 |
| Muscle spasms | 3 (5.9) | 0 | 0 | 0 |
| Pain in extremity | 3 (5.9) | 0 | 0 | 0 |
| Bone pain | 2 (3.9) | 0 | 1 (2.0) | 0 |
| Flank pain | 2 (3.9) | 0 | 0 | 0 |
| Muscular weakness | 2 (3.9) | 1 (2.0) | 0 | 0 |
| Spinal pain | 2 (3.9) | 0 | 0 | 0 |
| Joint range of motion decreased | 1 (2.0) | 0 | 0 | 0 |
| Monoarthritis | 1 (2.0) | 0 | 0 | 0 |
| Soft tissue necrosis | 1 (2.0) | 0 | 0 | 0 |
| Respiratory, thoracic, and mediastinal disorders | | | | |
| Cough | 10 (9.6) | 0 | 1 (2.0) | 0 |
| Dyspnea | 6 (11.8) | 3 (5.9) | 0 | 0 |
| Dyspnea exertional | 4 (7.8) | 0 | 1 (2.0) | 0 |
| Pleural effusion | 3 (5.9) | 1 (2.0) | 0 | 0 |
| Pneumonitis | 2 (3.9) | 0 | 2 (3.9) | 0 |
| Allergic sinusitis | 1 (2.0) | 0 | 0 | 0 |
| Hiccups | 1 (2.0) | 0 | 0 | 0 |
| Lung infiltration | 1 (2.0) | 0 | 1 (2.0) | 0 |
| Nasal congestion | 1 (2.0) | 0 | 0 | 0 |
| Oropharyngeal pain | 1 (2.0) | 0 | 0 | 0 |
| Pulmonary hemorrhage | 1 (2.0) | 1 (2.0) | 0 | 0 |
| Pulmonary hypertension | 1 (2.0) | 0 | 0 | 0 |
| Rhinorrhea | 1 (2.0) | 0 | 0 | 0 |
| Upper airway cough syndrome | 1 (2.0) | 0 | 0 | 0 |
| Infections and infestations | | | | |
| Urinary tract infection | 6 (11.8) | 0 | 0 | 0 |
| Nasopharyngitis | 3 (5.9) | 0 | 0 | 0 |
| Folliculitis | 2 (3.9) | 0 | 0 | 0 |
| Oral candidiasis | 2 (3.9) | 0 | 0 | 0 |
| Upper respiratory tract infection | 2 (3.9) | 0 | 0 | 0 |
| Body tinea | 1 (2.0) | 0 | 0 | 0 |
| Bronchitis | 1 (2.0) | 0 | 0 | 0 |
| Cellulitis | 1 (2.0) | 1 (2.0) | 0 | 0 |
| Cystitis | 1 (2.0) | 0 | 0 | 0 |
| Herpes virus infection | 1 (2.0) | 0 | 0 | 0 |
| Herpes zoster | 1 (2.0) | 0 | 0 | 0 |
| Labyrinthitis | 1 (2.0) | 0 | 0 | 0 |
| Localized infection | 1 (2.0) | 0 | 0 | 0 |
| Esophageal candidiasis | 1 (2.0) | 0 | 0 | 0 |
| Osteomyelitis | 1 (2.0) | 0 | 0 | 0 |
| Otitis media | 1 (2.0) | 0 | 0 | 0 |
| Pneumonia | 1 (2.0) | 1 (2.0) | 0 | 0 |
| Rash pustular | 1 (2.0) | 0 | 1 (2.0) | 0 |

| | | | | |
|---|---|---|---|---|
| Tinea versicolor | 1 (2.0) | 0 | 0 | 0 |
| Trichomoniasis | 1 (2.0) | 0 | 0 | 0 |
| Wound infection | 1 (2.0) | 0 | 0 | 0 |
| Skin and subcutaneous disorders | | | | |
| Pruritus | 5 (9.8) | 0 | 2 (3.9) | 0 |
| Erythema | 3 (5.9) | 0 | 2 (3.9) | 0 |
| Rash | 3 (5.9) | 0 | 2 (3.9) | 0 |
| Dry skin | 2 (3.9) | 0 | 1 (2.0) | 0 |
| Night sweats | 2 (3.9) | 0 | 0 | 0 |
| Rash maculopapular | 2 (3.9) | 0 | 2 (3.9) | 0 |
| Rash pruritic | 2 (3.9) | 0 | 1 (2.0) | 0 |
| Actinic keratosis | 1 (2.0) | 0 | 0 | 0 |
| Dermatitis contact | 1 (2.0) | 0 | 0 | 0 |
| Hyperhidrosis | 1 (2.0) | 0 | 0 | 0 |
| Photosensitivity reaction | 1 (2.0) | 0 | 1 (2.0) | 0 |
| Rash macular | 1 (2.0) | 0 | 0 | 0 |
| Skin burning sensation | 1 (2.0) | 0 | 1 (2.0) | 0 |
| Skin hyperpigmentation | 1 (2.0) | 1 (2.0) | 0 | 0 |
| Skin lesion | 1 (2.0) | 0 | 0 | 0 |
| Skin ulcer | 1 (2.0) | 1 (2.0) | 0 | 0 |
| Vitiligo | 1 (2.0) | 0 | 1 (2.0) | 0 |
| Investigations | | | | |
| Weight decreased | 9 (17.6) | 0 | 1 (2.0) | 0 |
| Aspartate aminotransferase increased | 3 (5.9) | 0 | 3 (5.9) | 0 |
| Activated partial thromboplastin time prolonged | 2 (3.9) | 2 (3.9) | 0 | 0 |
| Alanine aminotransferase increased | 2 (3.9) | 0 | 2 (3.9) | 0 |
| Blood alkaline phosphatase increased | 2 (3.9) | 0 | 1 (2.0) | 0 |
| Blood lactate dehydrogenase increased | 2 (3.9) | 1 (2.0) | 0 | 0 |
| Gamma-glutamyltransferase increased | 2 (3.9) | 2 (3.9) | 1 (2.0) | 1 (2.0) |
| Ammonia increased | 1 (2.0) | 0 | 0 | 0 |
| Blood bilirubin increased | 1 (2.0) | 0 | 1 (2.0) | 0 |
| Blood thyroid stimulating hormone increased | 1 (2.0) | 0 | 1 (2.0) | 0 |
| Body temperature increased | 1 (2.0) | 0 | 1 (2.0) | 0 |
| C-reactive protein increased | 1 (2.0) | 0 | 1 (2.0) | 0 |
| Electrocardiogram QT prolonged | 1 (2.0) | 1 (2.0) | 0 | 0 |
| International normalized ratio increased | 1 (2.0) | 0 | 0 | 0 |
| Lipase increased | 1 (2.0) | 1 (2.0) | 1 (2.0) | 1 (2.0) |
| Vitamin $B_{12}$ decreased | 1 (2.0) | 0 | 0 | 0 |
| Injury, poisoning, and procedural complications | | | | |
| Infusion-related reaction | 13 (25.5) | 0 | 13 (25.5) | 0 |
| Fall | 2 (3.9) | 0 | 0 | 0 |
| Chest injury | 1 (2.0) | 0 | 0 | 0 |
| Contusion | 1 (2.0) | 0 | 0 | 0 |
| Joint dislocation | 1 (2.0) | 0 | 0 | 0 |
| Joint injury | 1 (2.0) | 0 | 0 | 0 |
| Radiation skin injury | 1 (2.0) | 0 | 0 | 0 |
| Stoma site pain | 1 (2.0) | 0 | 0 | 0 |

| | | | | |
|---|---|---|---|---|
| Metabolism and nutrition disorders | | | | |
| Decreased appetite | 9 (17.6) | 1 (2.0) | 1 (2.0) | 0 |
| Hypokalemia | 6 (11.8) | 3 (5.9) | 1 (2.0) | 1 (2.0) |
| Hypophosphatemia | 3 (5.9) | 1 (2.0) | 0 | 0 |
| Dehydration | 2 (3.9) | 1 (2.0) | 0 | 0 |
| Hyperphosphatemia | 2 (3.9) | 0 | 1 (2.0) | 0 |
| Hyperuricemia | 2 (3.9) | 1 (2.0) | 0 | 0 |
| Hyponatremia | 2 (3.9) | 1 (2.0) | 0 | 0 |
| Gout | 1 (2.0) | 0 | 0 | 0 |
| Hypercalcemia | 1 (2.0) | 0 | 0 | 0 |
| Hyperglycemia | 1 (2.0) | 1 (2.0) | 0 | 0 |
| Hypertriglyceridemia | 1 (2.0) | 1 (2.0) | 0 | 0 |
| Hypoglycemia | 1 (2.0) | 1 (2.0) | 0 | 0 |
| Hypomagnesemia | 1 (2.0) | 0 | 0 | 0 |
| Nervous system disorders | | | | |
| Dysgeusia | 6 (11.8) | 0 | 4 (7.8) | 0 |
| Dizziness | 4 (7.8) | 0 | 1 (2.0) | 0 |
| Headache | 4 (7.8) | 0 | 2 (3.9) | 0 |
| Carpal tunnel syndrome | 2 (3.9) | 0 | 0 | 0 |
| Dysarthria | 2 (3.9) | 0 | 0 | 0 |
| Peripheral sensory neuropathy | 2 (3.9) | 0 | 0 | 0 |
| Somnolence | 2 (3.9) | 0 | 0 | 0 |
| Dyskinesia | 1 (2.0) | 0 | 0 | 0 |
| Hypoesthesia | 1 (2.0) | 0 | 0 | 0 |
| Paresthesia | 1 (2.0) | 0 | 0 | 0 |
| Polyneuropathy | 1 (2.0) | 0 | 0 | 0 |
| Restless legs syndrome | 1 (2.0) | 0 | 0 | 0 |
| Seizure | 1 (2.0) | 0 | 0 | 0 |
| Sensorimotor disorder | 1 (2.0) | 0 | 0 | 0 |
| Syncope | 1 (2.0) | 1 (2.0) | 0 | 0 |
| Blood and lymphatic system disorders | | | | |
| Anemia | 7 (13.7) | 2 (3.9) | 1 (2.0) | 0 |
| Lymphopenia | 3 (5.9) | 1 (2.0) | 2 (3.9) | 0 |
| Iron deficiency anemia | 2 (3.9) | 0 | 0 | 0 |
| Increased tendency to bruise | 1 (2.0) | 0 | 0 | 0 |
| Leukopenia | 1 (2.0) | 0 | 1 (2.0) | 0 |
| Psychiatric disorders | | | | |
| Insomnia | 4 (7.8) | 0 | 1 (2.0) | 0 |
| Anxiety | 2 (3.9) | 0 | 0 | 0 |
| Affective disorder | 1 (2.0) | 0 | 0 | 0 |
| Agitation | 1 (2.0) | 0 | 0 | 0 |
| Confusional state | 1 (2.0) | 0 | 0 | 0 |
| Delirium | 1 (2.0) | 0 | 0 | 0 |
| Depressed mood | 1 (2.0) | 0 | 0 | 0 |
| Depression | 1 (2.0) | 0 | 1 (2.0) | 0 |
| Depressive symptom | 1 (2.0) | 0 | 0 | 0 |
| Mental status changes | 1 (2.0) | 1 (2.0) | 0 | 0 |
| Restlessness | 1 (2.0) | 0 | 0 | 0 |

| | | | | |
|---|---|---|---|---|
| Renal and urinary disorders | | | | |
| Hematuria | 3 (5.9) | 0 | 0 | 0 |
| Acute kidney injury | 2 (3.9) | 1 (2.0) | 0 | 0 |
| Dysuria | 2 (3.9) | 0 | 0 | 0 |
| Bladder dilatation | 1 (2.0) | 0 | 0 | 0 |
| Bladder trabeculation | 1 (2.0) | 0 | 0 | 0 |
| Pollakiuria | 1 (2.0) | 0 | 0 | 0 |
| Proteinuria | 1 (2.0) | 0 | 0 | 0 |
| Urinary retention | 1 (2.0) | 0 | 0 | 0 |
| Urobilinuria | 1 (2.0) | 0 | 0 | 0 |
| Cardiac disorders | | | | |
| Atrial fibrillation | 2 (3.9) | 0 | 0 | 0 |
| Acute coronary syndrome | 1 (2.0) | 1 (2.0) | 0 | 0 |
| Cardiac arrest | 1 (2.0) | 1 (2.0) | 0 | 0 |
| Conduction disorder | 1 (2.0) | 0 | 0 | 0 |
| Myocardial infarction | 1 (2.0) | 1 (2.0) | 0 | 0 |
| Pericardial effusion | 1 (2.0) | 0 | 0 | 0 |
| Tachycardia | 1 (2.0) | 0 | 1 (2.0) | 0 |
| Neoplasms benign, malignant, and unspecified (including cysts and polyps) | | | | |
| Cancer pain | 2 (3.9) | 0 | 0 | 0 |
| Metastasis | 2 (3.9) | 0 | 0 | 0 |
| Seborrheic keratosis | 2 (3.9) | 0 | 0 | 0 |
| Tumor hemorrhage | 2 (3.0) | 0 | 0 | 0 |
| Metastases to muscle | 1 (2.0) | 0 | 0 | 0 |
| Metastatic malignant melanoma | 1 (2.0) | 0 | 0 | 0 |
| Tumor-associated fever | 1 (2.0) | 0 | 0 | 0 |
| Tumor ulceration | 1 (2.0) | 0 | 0 | 0 |
| Vascular disorders | | | | |
| Hypertension | 3 (5.9) | 3 (5.9) | 0 | 0 |
| Hypotension | 1 (2.0) | 0 | 0 | 0 |
| Hypovolemic shock | 1 (2.0) | 0 | 0 | 0 |
| Peripheral coldness | 1 (2.0) | 0 | 0 | 0 |
| Ear and labyrinth disorders | | | | |
| Ear pain | 1 (2.0) | 0 | 0 | 0 |
| Ear pruritus | 1 (2.0) | 0 | 0 | 0 |
| Hypoacusis | 1 (2.0) | 0 | 0 | 0 |
| Vertigo | 1 (2.0) | 0 | 0 | 0 |
| Endocrine disorders | | | | |
| Hypothyroidism | 3 (5.9) | 0 | 2 (3.9) | 0 |
| Hyperparathyroidism primary | 1 (2.0) | 0 | 1 (2.0) | 0 |
| Hyperthyroidism | 1 (2.0) | 0 | 1 (2.0) | 0 |
| Eye disorders | | | | |
| Conjunctival hemorrhage | 1 (2.0) | 0 | 0 | 0 |
| Glaucoma | 1 (2.0) | 0 | 0 | 0 |
| Visual acuity reduced | 1 (2.0) | 0 | 0 | 0 |
| Hepatobiliary disorders | | | | |
| Hepatic pain | 2 (3.9) | 0 | 0 | 0 |
| Cholecystitis | 1 (2.0) | 1 (2.0) | 0 | 0 |
| Hepatomegaly | 1 (2.0) | 1 (2.0) | 0 | 0 |

| | | | | |
|---|---|---|---|---|
| Immune system disorders | | | | |
| Drug hypersensitivity | 1 (2.0) | 0 | 1 (2.0) | 0 |
| Hypersensitivity | 1 (2.0) | 0 | 1 (2.0) | 0 |
| Sarcoidosis | 1 (2.0) | 0 | 1 (2.0) | 0 |
| Reproductive system and breast disorders | | | | |
| Benign prostatic hyperplasia | 1 (2.0) | 0 | 0 | 0 |
| Breast pain | 1 (2.0) | 0 | 1 (2.0) | 0 |
