# Supplementary material for: Avelumab in patients with previously treated metastatic melanoma: phase 1b results from the JAVELIN Solid Tumor trial
Source: J Immunother Cancer. 2019 Jan 16;7:12. doi: 10.1186/s40425-018-0459-y (PMC6335739; doi:10.1186/s40425-018-0459-y)
Supplement: Supplementary file 7 — Table S4. All serious adverse events (N = 51). (PDF) (PDF 101 kb) [file 40425_2018_459_MOESM7_ESM.pdf]

Additional file 7: Table S4. All serious adverse events (N=51).

**Primary system organ class**<br>**MedDRA preferred term**	**All causality, n (%)**
Patients with ≥1 event	24 (47.1)
General disorders and administration site conditions	
Disease progression	3 (5.9)
Pyrexia	3 (5.9)
Asthenia	2 (3.9)
General physical health deterioration	1 (2.0)
Cardiac disorders	
Atrial fibrillation	2 (3.9)
Cardiac arrest	1 (2.0)
Myocardial infarction	1 (2.0)
Gastrointestinal disorders	
Gastrointestinal hemorrhage	2 (3.9)
Vomiting	2 (3.9)
Nausea	1 (2.0)
Esophagitis	1 (2.0)
Metabolism and nutrition disorders	
Dehydration	1 (2.0)
Hyperglycemia	1 (2.0)
Hyperuricemia	1 (2.0)
Hypoglycemia	1 (2.0)
Respiratory, thoracic, and mediastinal disorders	
Dyspnea	1 (2.0)
Pleural effusion	1 (2.0)
Pulmonary hemorrhage	1 (2.0)
Infections and infestations	
Cellulitis	1 (2.0)
Pneumonia	1 (2.0)
Injury, poisoning, and procedural complications	
Fall	1 (2.0)
Infusion-related reaction	1 (2.0)
Neoplasms benign, malignant, and unspecified (including cysts and polyps)	
Metastasis	2 (3.9)
Nervous system disorders	
Sensorimotor disorder	1 (2.0)
Syncope	1 (2.0)
Renal and urinary disorders	
Acute kidney injury	1 (2.0)
Hematuria	1 (2.0)
Blood and lymphatic system disorders	
Anemia	1 (2.0)
Hepatobiliary disorders	
Cholecystitis	1 (2.0)
Immune system disorders	
Sarcoidosis	1 (2.0)

Psychiatric disorders<br>Mental status changes	1 (2.0)
Skin and subcutaneous disorders<br>Skin ulcer	1 (2.0)
